# Evidence for passerine bird pollination in *Rhododendron* species

**DOI:** 10.1093/aobpla/plx062

**Published:** 2017-11-09

**Authors:** Zhi-Huan Huang, Yun-Peng Song, Shuang-Quan Huang

**Affiliations:** Guangxi Institute of Botany, Guangxi Zhuang Autonomous Region and Chinese Academy of Sciences, Guilin, China; Institute of Evolution and Ecology, School of Life Sciences, Central China Normal University, Wuhan, China

**Keywords:** Bee pollination, bird pollination, flower size, fruit/seed production, low temperature, pollinator exclusion experiment, *Rhododendron*

## Abstract

When insect activity is limited at low temperature, birds may be comparatively more important pollinators than insects for flowering plants. It has been thought that many large-flowered *Rhododendron* species are pollinated by local birds in the Himalayan regions because most of these species flower in spring at high elevation with cool atmospheric temperature. However, experimental evidence for the role of bird pollination in this hyperdiverse genus remains scarce. To examine the role of birds and insects in pollination, we observed floral visitors to 15 *Rhododendron* species with different floral sizes and abundant flowering individuals in the eastern Himalayas, Southwest China. To examine the role of birds and insects in female reproductive success in each species, cages were used to exclude birds but not insects from visiting flowers and net bags were used to exclude all floral visitors. Inflorescences where visitation was excluded did not produce fruits in any of the *Rhododendron* species, indicating that sexual reproduction in these species depended on pollinator visitation. Bird visits were generally less frequent than bee visits in the studied species. However, in the nine species on which bird visitors were observed, fruit and/or seed set were greatly reduced in inflorescences caged to exclude birds but not bees, compared to open-pollinated inflorescences. In the other six species on which bird visitation was not observed, fruit and seed set did not differ significantly between caged and open inflorescences except in one species (*R. wardii*). Manipulations to achieve selective exclusion of visitors demonstrated that birds could be effective pollinators for 10 out of 15 studied *Rhododendron* species in the eastern Himalayas. Floral characteristics of these *Rhododendron* species and weather conditions might favour the evolution of bird pollination systems in the East Himalayas.

## Teaser

It has been hypothesized that flowering plants at high elevation could be pollinated by local passerine birds given that low temperature limits insect activity. In particular, species with large flowers are likely to be pollinated by large-body animals because a morphological fit between floral morphology and pollinator body could facilitate pollen transfer. Huang et al. investigated pollinator species and their visitation frequency in 15 Rhododendron species from East Himalayas. The pollinator experiments excluding all pollinators, or just birds in the 15 species demonstrated that birds acted as pollinators in 2/3 studied Rhododendron species, in support of the hypotheses.

## Introduction

It has been estimated that 87.5 % of flowering plant species rely on animals for pollination (Ollerton *et al.* 2011). Most pollinators are insects but birds are important pollinators for some species in about 65 plant families (Cronk and Ojeda 2008). Previous comparative studies have shown that shifts from bee pollination to bird pollination have occurred independently in numerous lineages of flowering plants (Kay *et al.* 2005; Specht 2006; Whittall and Hodges 2007; Wilson *et al.* 2007; Sakai *et al.* 2013), whereas reversal is much less common (Wolfe *et al.* 2006; Wilson *et al.* 2007). Of these two groups of pollinators, birds tend to be larger and can be more active in cool temperature than insects. It has been proposed that bird pollination enhances plant reproductive success at low atmospheric temperature when weather conditions are unfavourable for bees (Cruden 1972).

One hypothesis for the shift from bee to bird pollination is the high ratio of pollen receipt to pollen removal (Wilson *et al.* 2007). A successful pollination depends on a vector removing pollen from one flower and delivering pollen to another flower. Unlike bird foraging behaviour, which involves little grooming, bees (e.g. bumblebees) usually groom pollen from their body into corbiculae where it is unavailable for pollen transfer to the next flower visited. Empirical studies have shown that pollinators that rarely exhibit grooming behaviour are more efficient at delivering pollen (Muchhala and Thomson 2010). In addition, birds are considered to become important supplemental pollinators in habitats where insect activity is limited. For example, high mountains, where cold and/or rainy weather conditions are frequent, are less conducive to insect activity (Stiles 1971, 1978; Cruden 1972). Furthermore, insects are generally short-lived and overwinter as immature stages or in hibernation (i.e. many bees, see Goulson 2003), and are rarely available for plants that flower in winter or early spring when the temperature is low.

*Rhododendron* (Ericaceae) is one of the largest plant genera with around 1000 evergreen or deciduous species mainly in Asia (Chamberlain *et al.* 1996), including many endemic species in the Himalayan region. Most species in this region have conspicuous flowers. *Rhododendron* species usually blossom from late winter to early summer when the temperature is low and the activity of insects is restricted. *Rhododendron* can be a model genus in which to examine the divergent evolution of floral traits related to pollinator shifts because the flower morphology varies widely in the same region. In diverse plant groups, selection on floral morphology has been shown to be mediated in part by morphometric interactions with the mouth or body part length of pollinators across regional floras (Inouye 1980; Inouye and Pyke 1988; Nilsson 1988; Alexandersson and Johnson 2002; Moré *et al.* 2012). Based on morphological studies of nearly 300 tropical species from the Malayan region (Southeast Asia to Northern Australia), Stevens (1976) estimated that one-third of *Rhododendron* species may have evolved bird pollination, particularly large-flowered species at high elevations. Although insects have been observed to be effective pollinators in several *Rhododendron* species (Stout *et al.* 2006; Stout 2007; Tagane *et al.* 2008; Ma *et al.* 2010, 2015; Kudo *et al.* 2011; Epps *et al.* 2015), anecdotal observations showed that sunbirds and passeriforms feed on nectar in numerous species (Huang 2011; Georgian *et al.* 2015). However, there has been no experimental examination of the effectiveness of bird pollination in this well-known genus.

To test the effectiveness of birds and insects as pollinators of 15 *Rhododendron* species, we addressed the following specific questions: (i) Given that low temperature limits insect activity, do warm-blooded birds act as floral visitors in some *Rhododendron* species? (ii) How do birds contribute to pollination in these species? (iii) Given that a morphological fit between floral morphology and pollinator body could facilitate pollen transfer (see Stevens 1976), do birds tend to visit relatively large *Rhododendron* flowers? We investigated pollinator species and their visitation frequency in 15 *Rhododendron* species that had abundant flowering individuals with a wide variation in flower size. We conducted two types of pollinator experiments by excluding all pollinators, or just birds, to compare the pollination role of birds and insects in these *Rhododendron* species.

## Methods

### Species and study area

*Rhododendron* (Ericaceae) is a species-rich genus of perennial shrubs or trees. Because their flowers are usually large and brightly coloured and flower in spring, *Rhododendron* species are important horticultural plants worldwide. The diversity of *Rhododendron* is especially high in East Asia, with over 500 species endemic to Southwest China. Floral and vegetative traits are highly variable, but *Rhododendron* flowers are characterized by an open-shaped funnelform, campanulate or tubular corolla which is formed by petal fusion. There are usually 5–10 stamens surrounding one exserted style with a capitate stigma. Pollen grains released from the poricidal anthers are connected together by sticky viscin threads.

The field survey was conducted in Cangshan National Nature Reserve (N 25°43′; E 100°01′) and Laojunshan Nature Reserve (N 26°39′; E 99°44′), Yunnan Province, Southwest China. These areas are in the Hengduan Mountains, East Himalayas, with elevations from 1800 to 4200 m (see details in Table 1), usually comprising a mosaic of deciduous broad-leaved and coniferous forests, pastures and scrubby stream mountain vegetation. We investigated floral traits of 15 *Rhododendron* species in which abundant flowering individuals were available and conducted pollinator selective exclusion experiments on these species.

**Table 1.** Details of field populations of 15 *Rhododendron* species, pollinator observation censuses and species of bird visitors that were observed in Cangshan and Laojunshan Mountains, Yunnan Province, Southwest China. The two sunbirds are *Aethopyga gouldiae* and *A. ignicauda* (Nectariniidae).

Species	Life form	Location	Latitude (N)	Longitude (E)	Altitude (m)	Flowering time	Observed 0.5-h censuses	Bird visitor species
*R. beesianum*	Small trees	Laojunshan	26°39′23″	99°44′23″	3300–4000	May–June	23	*Aethopyga gouldiae* (Nectariniidae), *Phylloscopus affinis* (Phylloscopidae), *Zosterops japonicus* (Zosteropidae)
*R. clementinae*	Shrubs	Laojunshan	26°39′67″	99°44′45″	3300–4200	May–June	18	*A. gouldiae*, *P. affinis*
*R. cyanocarpum*	Small trees	Cangshan	25°67′41″	100°10′71″	3400–4000	April–May	20	*P. affinis*, *Pycnonotus jocosus monticola* (Pycnonotidae)
*R. delavayi*	Small trees	Cangshan	25°82′41″	99°98′43″	2200–2500	March–May	20	*Heterophasia melanoleuca* (Leiothrichidae), *P. jocosus monticola*
*R. neriiflorum*	Shrubs	Cangshan	25°42′25″	100°04′38″	2550–3600	April–May	20	*A. ignicauda*, *P. jocosus monticola*
*R. lacteum*	Small trees	Cangshan	25°66′86″	100°09′23″	3500–4050	April–May	20	*A. ignicauda*, *P. jocosus monticola*
*R. oreotrephes*	Shrubs	Laojunshan	26°54′16″	99°76′32″	3300–3700	May–July	18	*A. gouldiae*, *P. affinis*
*R. rex*	Small trees	Cangshan	25°70′12″	100°08′79″	3200–3800	May–June	20	*A. ignicauda*, *P. affinis*
*R. sinogrande*	Trees	Cangshan	25°82′57″	99°98′06″	2100–2600	April–May	24	*A. ignicauda*, *Yuhina occipitalis* (Zosteropidae)
*R. wardii*	Small trees	Laojunshan	26°56′52″	99°61′36″	3000–3800	June–July	29	—
*R. microphyton*	Shrubs	Cangshan	25°81′54″	100°08′46″	2200–2800	March–May	18	—
*R. racemosum*	Shrubs	Cangshan	25°82′36″	100°08′43″	2100–3500	March–May	24	—
*R. simsii*	Shrubs	Cangshan	25°82′13″	100°09′28″	1800–2700	April–May	24	—
*R. trichocladum*	Shrubs	Cangshan	25°41′65″	100°06′42″	3200–3600	May–June	16	—
*R. virgatum*	Shrubs	Cangshan	25°81′50″	100°08′20″	2200–2800	March–May	26	—

### Pollinator observations

To quantify the visiting frequency of pollinators to these species, we conducted systematic bird and insect censuses on clear days (no rain) from April to June in 2013, for a total of 160 h (Table 1). We observed pollinator visits in at least 10 half-hour censuses in each species with the aid of binoculars. For each *Rhododendron* species, between early morning (0800 h) and late afternoon (1800 h), we haphazardly selected at least three flowering inflorescences on three different individuals within a distance of 5–10 m containing 60–400 flowers for pollinator observations. Only visitors that contacted anthers and stigmas were recorded. Visitation frequency was obtained as the mean number of visits per flower per hour for each visitor species.

### Pollinator exclusion experiment

**Figure 1.**
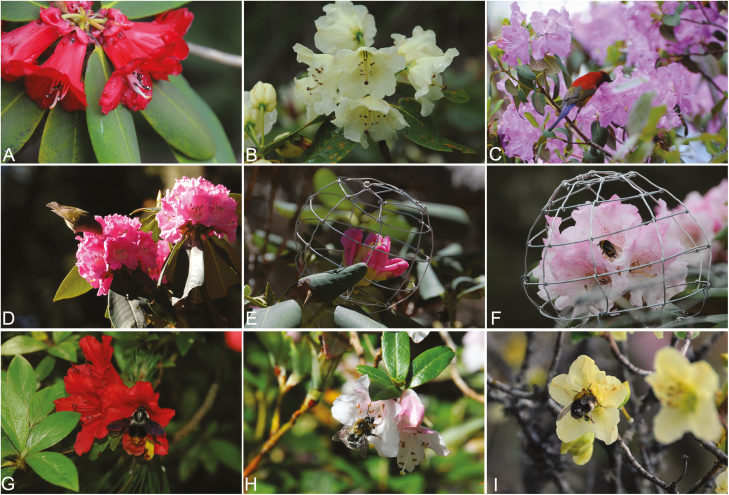
Inflorescences of nine *Rhododendron* species showing diverse floral morphology, floral visitors and caged treatments for bird exclusion. (A) Crimson tubular corolla of *R. neriiflorum*; (B) bright yellow campanulate corolla of *R. wardii*; (C) sunbird (*Aethopyga gouldiae*) visiting open-shaped flowers of *R. oreotrephes*; (D) *Phylloscopus affinis* (Sylviidae) sucking nectar from *R. beesianum*; (E) caged inflorescence in *R. cyanocarpum*; (F) a bumblebee (*Bombus friseanus* Skorikov) visiting caged *R. clementina* flowers from which birds were excluded; (G) *Bombus avanus* visiting flowers of *R. simsii*; (H) *Bombus hypnorum* visiting flowers of *R. trichocladum*; (I) *Bombus festivus* visiting flowers of *R. virgatum*.

To evaluate the role in pollination of birds and insects, we conducted two types of pollinator exclusions, caged and bagged, during early April and late May 2013. On 6–12 flowering individuals of each species, flower buds on three inflorescences per plant were randomly selected for the following treatments: (i) exclusion of all potential visitors using small-mesh (0.33 × 0.33 mm) nylon nets; (ii) exclusion of bird visitors using large-mesh (around 30 × 25 mm) metal cages made with aluminium wire permitting insects to access floral rewards (cages were placed around flower buds and fixed to the supporting stems; Fig. 1C); and (iii) natural pollination in which unmanipulated inflorescences were open to all floral visitors. To examine whether the cages interfered with the foraging behaviour of insects, we observed visitors to the caged and open (unmanipulated) inflorescences of four species at the beginning of the inflorescence manipulations. The behaviour of bees on caged inflorescences was similar to that on the open inflorescences in *R. beesianum*, *R. clementinae*, *R. cyanocarpum* and *R. lacteum*. These initial investigations indicated that our bird-cage treatments were effectively excluding bird visits but not insects (see also Kunitake *et al.* 2004; Botes *et al.* 2009; Fang *et al.* 2012). We labelled 6–12 flower buds in each inflorescence with plastic tags and cotton threads (one inflorescence usually contained 10–20 flowers), so that each treatment involved more than 50 labelled flowers per species. In September when fruits matured, we collected all fruits that were tagged from infructescences. Matured and aborted seeds (which were basically undeveloped ovules with obviously smaller than the developed seeds) per fruit were counted (>25 fruits were obtained from each treatment per species). Seed set per fruit was number of matured seeds divided by total number of ovules (i.e. matured + aborted seeds).

### Measurement of flower size

To examine whether flower size relates to pollinator type, we measured the floral opening diameter and corolla-tube length of 15 *Rhododendron* species from early April to middle June in 2012 and 2013. The maximum and minimum separation distance of two opposite petal tips were measured to calculate the mean floral opening diameter. The corolla-tube length was measured from the base of the ovary to the top of the corolla gap. Thirty to 50 flowers per species (3–5 flowers from each of 10 randomly chosen plants) were measured using an electronic digital caliper to an accuracy of 0.01 mm.

### Data analysis

The following generalized linear model (GLM) analyses were conducted by the glm function in R version 3.1.0 (http://cran.r-project.org/). The difference in visitation frequency between birds and insects within each *Rhododendron* species with bird pollination was compared with Gaussian distribution and log-link function. Differences in fruit set (the ratio of fruits/flowers) and seed set (seeds/ovules) under the three pollination treatments per species were analysed with binomial and Gaussian distribution and logit link function. Differences in flower size and elevation with pollinator types among 15 species were analysed to estimate whether bird-pollinated flowers are larger and appear at higher elevation than insect-pollinated flowers, with Gaussian distribution and identity link function. To examine whether shrub *Rhododendron* species appear in higher elevation than the tree species, life forms of the 15 species were recorded and mean values of elevation were compared with Gaussian distribution and identity link function.

## Results

### Pollinator types and abundance

Bees (including bumblebees and honeybees) were observed visiting flowers in all 15 *Rhododendron* species. *Bombus avanus*, *B. festivus*, *B. friseanus* and *B. hypnorum* were the four most frequent bumblebees (Fig. 1F–I), while *Apis cerana* was the most frequent honeybee. To access nectar at the base of flowers, bees usually landed towards the upper region of the corolla where there was a visible honey guide. Bee visitation frequency ranged from 0.04 (in *R. sinogrande*) to 1.12 visits flower^−1^ h^−1^ (*R. virgatum*). Bird visitation was observed in nine *Rhododendron* species which generally had large flowers (Tables 1 and 2). On the other six species, we observed no birds but only insect visitation. Interestingly, in the large-flowered *R. wardii* bird visitation was not observed but pollinator exclusion experiments suggested birds serving as pollinators (see below). Butterfly and moth visitors to these *Rhododendron* flowers were generally rare. However, in *R. simsii* we observed 48 visits by a butterfly *Pachliopta aristolochiae* in 24 observation censuses and 74 visits to *R. virgatum* by a hawkmoth *Macroglossum pyrrhosticta* in 26 observation censuses.

**Table 2.** Flower size, colour and visit frequency (mean ± SE) of two types of floral visitors in 15 *Rhododendron* species.

Plant species	Flower colour	Opening diameter (mm)	Tube length (mm)	Bird visit frequency	Bee visit frequency
*R. beesianum*	White or pink	48.30 ± 0.63	36.88 ± 0.37	0.11 ± 0.03	0.13 ± 0.04
*R. clementinae*	Rosaceous to pink	51.75 ± 0.60	33.07 ± 0.65	0.08 ± 0.02	0.49 ± 0.10
*R. cyanocarpum*	White to pink	52.28 ± 0.79	33.57 ± 0.46	0.12 ± 0.03	0.05 ± 0.02
*R. delavayi*	Deep crimson to carmine	48.14 ± 1.02	34.02 ± 0.65	0.06 ± 0.02	0.22 ± 0.05
*R. neriiflorum*	Crimson or bright red	33.28 ± 0.98	24.69 ± 0.64	0.14 ± 0.04	0.17 ± 0.06
*R. lacteum*	Pure yellow	49.13 ± 0.74	36.11 ± 0.56	0.05 ± 0.02	0.38 ± 0.10
*R. oreotrephes*	Pale red or purplish red	32.75 ± 0.49	14.88 ± 0.20	0.15 ± 0.04	1.02 ± 0.11
*R. rex*	Creamy white to pink	49.47 ± 0.86	37.48 ± 0.39	0.13 ± 0.05	0.85 ± 0.21
*R. sinogrande*	Creamy white to pale yellow	38.83 ± 0.68	41.80 ± 0.67	0.15 ± 0.04	0.04 ± 0.01
*R. wardii*	Bright yellow	54.38 ± 0.86	19.26 ± 0.24	0	0.69 ± 0.08
*R. microphyton*	Pale purple or light purplish red	20.28 ± 0.49	12.13 ± 0.20	0	0.64 ± 0.12
*R. racemosum*	Pink or purple	21.23 ± 0.26	7.81 ± 0.20	0	0.55 ± 0.11
*R. simsii*	Rose, bright to dark red	46.58 ± 0.67	25.33 ± 0.45	0	0.81 ± 0.11
*R. trichocladum*	Yellow or greenish yellow	27.09 ± 0.30	11.36 ± 0.14	0	1.00 ± 0.15
*R. virgatum*	Light red or white	28.40 ± 0.31	12.72 ± 0.25	0	1.12 ± 0.17

In the nine *Rhododendron* species with bird visitation, one species usually involved two or three bird visitor species (Table 1). Two sunbird species *Aethopyga gouldiae* and *A. ignicauda* (Nectariniidae), respectively, visited three and four *Rhododendron* species. The red-whiskered bulbul *Pycnonotus jocosus monticola* (Pycnonotidae) acted as floral visitors for four *Rhododendron* species in Cangshan Mountain (Table 1). The two sunbirds were the most frequent passerine visitors to five of the nine species, and other passerine visitors from the four bird families were also observed sucking nectar in five species (*R. beesianum*, *R. cyanocarpum*, *R. delavayi*, *R. rex* and *R. sinogrande*). For example, the visitation of *A. gouldiae* accounted for 92.1 % of the total bird visits (*n* = 214) in *R. oreotrephes* (Fig. 1C). Dark-backed sibia *Heterophasia melanoleuca* (Leiothrichidae) was mainly observed in *R. delavayi*, accounting for 82.2 % of bird visits (101). Visitation by *P. jocosus monticola* accounted for 76.9 % of bird visits (168) to *R. cyanocarpum* (Fig. 1E). Visitation by *Yuhina occipitalis* (Zosteropidae) accounted for 83.2 % bird visits in *R. sinogrande* (465). Overall, visitation frequency of birds (0.12 ± 0.01 visits flower^−1^ h^−1^) was generally lower (estimate = 0.25, SE = 0.12, *t* = 2.12, *P* = 0.050) than that of bees (0.37 ± 0.12 visits flower^−1^ h^−1^) in the nine species with bird visitation (Table 2).

### Pollination roles of the two pollinator groups

Birds were not observed to approach the caged inflorescences in any species but flowers in cages were accessible to diverse insects. Nylon-net-bagged inflorescences did not produce fruits in any *Rhododendron* species, indicating that sexual reproduction in these species depended on pollinator visitation. Compared to open-pollinated inflorescences, the fruit set of caged flowers from which birds were excluded was significantly decreased in eight species but not in the five species with only bee visitation (Table 3). Only in two species in which bird visitation was observed (*R. cyanocarpum* and *R. lacteum*), fruit set was not significantly affected by the exclusion of birds. In all of the nine species on which bird visitation was observed, and in *R. wardii*, the seed set of caged inflorescences was significantly decreased. In the other five species, on which bird visitation was not observed, seed set did not differ significantly between caged and open-pollinated inflorescences (Table 4).

**Table 3.** Comparisons of fruit set (mean ± SE) between inflorescences caged to exclude birds and open-pollinated inflorescences in each of 15 *Rhododendron* species under a GLM with binomial distribution and logit link function. *P* values < 0.05 are in bold.

Species	Fruit set		Estimate	SE	*Z*	*P* value
	Open-pollinated	Caged				
*R. beesianum*	0.66 ± 0.02	0.22 ± 0.04	−1.08	0.18	−6.05	**<0.001**
*R. clementinae*	0.55 ± 0.05	0.37 ± 0.04	−0.40	0.17	−2.36	**0.02**
*R. cyanocarpum*	0.26 ± 0.04	0.21 ± 0.03	−0.12	0.24	−0.49	0.62
*R. delavayi*	0.37 ± 0.03	0.23 ± 0.04	−0.51	0.18	−2.94	**0.003**
*R. lacteum*	0.46 ± 0.05	0.36 ± 0.03	−0.24	0.13	−1.86	0.06
*R. neriiflorum*	0.92 ± 0.02	0.34 ± 0.05	−0.98	0.22	−4.56	**<0.001**
*R. oreotrephes*	0.86 ± 0.02	0.54 ± 0.04	−0.47	0.20	−2.27	**0.02**
*R. rex*	0.83 ± 0.03	0.49 ± 0.05	−0.55	0.14	−3.95	**<0.001**
*R. sinogrande*	0.37 ± 0.05	0.04 ± 0.01	−2.23	0.30	−7.46	**<0.001**
*R. wardii*	0.71 ± 0.04	0.38 ± 0.04	−0.63	0.19	−3.37	**<0.001**
*R. microphyton*	0.77 ± 0.04	0.70 ± 0.04	−0.10	0.20	−0.53	0.60
*R. racemosum*	0.86 ± 0.02	0.82 ± 0.03	−0.06	0.17	−0.36	0.72
*R. simsii*	0.67 ± 0.04	0.53 ± 0.04	−0.24	0.27	−0.90	0.37
*R. trichocladum*	0.90 ± 0.02	0.85 ± 0.04	−0.07	0.20	−0.38	0.71
*R. virgatum*	0.86 ± 0.02	0.79 ± 0.03	−0.09	0.19	−0.49	0.62

**Table 4.** Comparisons of seed set per fruit (mean ± SE) between inflorescences caged to exclude birds and open-pollinated inflorescences under a GLM with Gaussian distribution and identity link function in each of 15 *Rhododendron* species. *P* values < 0.05 are in bold.

Species	Seed set		Estimate	SE	*t*	*P* value
	Open-pollinated	Caged				
*R. beesianum*	0.59 ± 0.02	0.23 ± 0.04	−0.24	0.04	−5.96	**<0.001**
*R. clementinae*	0.46 ± 0.01	0.17 ± 0.02	−0.29	0.02	−12.88	**<0.001**
*R. cyanocarpum*	0.55 ± 0.03	0.25 ± 0.04	−0.23	0.05	−4.18	**<0.001**
*R. delavayi*	0.59 ± 0.01	0.10 ± 0.03	−0.37	0.04	−8.42	**<0.001**
*R. lacteum*	0.46 ± 0.02	0.26 ± 0.01	−0.19	0.03	−6.98	**<0.001**
*R. neriiflorum*	0.56 ± 0.03	0.23 ± 0.03	−0.32	0.04	−7.82	**<0.001**
*R. oreotrephes*	0.62 ± 0.02	0.36 ± 0.02	−0.26	0.03	−9.65	**<0.001**
*R. rex*	0.42 ± 0.03	0.11 ± 0.02	−0.28	0.04	−7.08	**<0.001**
*R. sinogrande*	0.41 ± 0.02	0.01 ± 0.01	−0.4	0.03	−15.54	**<0.001**
*R. wardii*	0.54 ± 0.02	0.41 ± 0.03	−0.16	0.04	−4.25	**<0.001**
*R. microphyton*	0.54 ± 0.03	0.52 ± 0.03	−0.02	0.04	−0.55	0.58
*R. racemosum*	0.65 ± 0.02	0.59 ± 0.02	−0.05	0.03	−1.5	0.14
*R. simsii*	0.53 ± 0.03	0.46 ± 0.03	−0.08	0.04	−1.8	0.08
*R. trichocladum*	0.73 ± 0.02	0.72 ± 0.03	−0.01	0.03	−0.41	0.69
*R. virgatum*	0.73 ± 0.02	0.70 ± 0.02	−0.03	0.03	−1.2	0.24

### Effects of flower size and pollinator groups

The elevation of seven tree species (mean ± SE, 3246 ± 625 m) was not significantly different (estimate = −0.001, SE = 0.001, *t* = −0.91, *P* = 0.38; Table 1) from that of the eight shrub species (2972 ± 544 m), indicating that life forms of the 15 *Rhododendron* species were not related to elevation. In the 10 species with potential bird pollination the diameter of the floral opening was significantly larger (45.83 ± 2.50 mm) than in the other five species (28.72 ± 4.74) (estimate = −17.12, SE = 4.83, *t* = −3.54, *P* = 0.004), while the corolla tube was significantly longer (31.18 ± 2.74 mm) than in the five species without bird pollination (13.87 ± 2.99) (estimate = −17.31, SE = 4.44, *t* = −3.90, *P* = 0.002). The 10 species in which seed set significantly decreased by exclusion of bird visits were distributed at relatively high elevation (mean ± SE, 3305 ± 172 m) compared to the five species on which only insect visits were observed (2690 ± 198 m) (estimate = −615.0, SE = −281.7, *t* = −2.18, *P* = 0.048) (Table 1). These results suggest that birds tended to visit relatively large *Rhododendron* flowers (Table 4) and distributed at higher elevations.

## Discussion

Our field observations and flower manipulations in 15 *Rhododendron* species indicated that passerine birds were likely to contribute to the pollination of 10 species, although bees were often the most frequent visitors. Our experimental exclusion of birds showed that fruit set and seed set were reduced by 46.9 % and 61.9 %, respectively, in the 10 *Rhododendron* species with bird visitation, while fruit/seed set of caged inflorescences did not decrease in the five *Rhododendron* species without bird visitation (Tables 3 and 4).

While birds, butterflies and sphingid moths were considered as probable pollinators for *Rhododendron* species (Ward 1937; Stevens 1976, 1985), bees have been reported to be major pollinators for *R. ponticum* (Stout *et al.* 2006; Stout 2007), *R. eriocapum* and *R. indicum* (Tagane *et al.* 2008), *R. cyanocarpum* and *R. delavyi* (Ma *et al.* 2010), *R. aureum* (Kudo *et al.* 2011) and *R. ferrugineum* (Milne and Abbott 2008). On the basis of floral traits it has been proposed that some *Rhododendron* species in tropical Malaysia were pollinated by bats (Stevens 1976, 1985; Danet 2012), but experimental evidence is not yet available. In the yellow-orange flowered *Rhododendron calendulaceum* which was flowering in summer at an elevation of 1160 m in southwestern Virginia, USA, Epps *et al.* (2015) observed two butterfly species and numerous bee species visiting the flowers, but only butterfly wings were the primary vehicle of pollination. In this species, bee visitors did not contact both anthers and stigmas; they functioned as either pollen or nectar robbers. Caging inflorescences to exclude butterflies resulted in almost complete fruit failure, demonstrating that butterflies were effective pollinators in this *Rhododendron* species. Anecdotal observations showed that three butterfly species, rather than bees, were major floral visitors in two populations of *R. indicum* (Tagane *et al.* 2008), and two bumblebee species, rather than butterflies, were effective pollinators in *R. cyanocarpum* in Yunnan Province (Ma *et al.* 2015). In the latter species we observed two bird species (*P. affinis* and *P. jocosus monticola*) acting as pollinators. We noted one butterfly species visiting *R. simsii* and it was a frequent visitor to *R. rubiginosum* in Laojunshan Mountains, Yunnan Province (Y.-P. Song *et al.*, Central China Normal University, unpubl. data), but further studies are needed to see whether the butterfly serves as an effective pollinator.

An investigation of floral visitors to six wild *Rhododendron* species in subtropical forest in Hong Kong, south China, showed that most insect visitors were large bees (*A. cerana*, *Bombus eximius* and *Xylocopa* spp.), hawkmoths and butterflies (Ng and Corlett 2000). The fork-tailed sunbird (*Aethopyga christinae*) was observed occasionally visiting the four *Rhododendron* species but bird contribution in pollination was neglected because of its low visitation rate (Ng and Corlett 2000). The four species had relatively larger flowers than the other two species without bird visitation based on their measurements of floral opening diameter and tube depth, consistent with our observation that large flowers are likely to evolve bird pollination. In a recent study of floral visitors with the aid of time-lapse camera trap observations in the red-flowered *Rhododendron floccigerum* Franchet in Yunnan Province, Georgian *et al.* (2015) proposed that four bird species (*A. gouldiae*, *Garrulax affinis*, *H. melanoleuca* and *Yuhina diademata*) and bumblebees were potential pollinators, given that birds’ heads and bees’ bodies were fully immersed in the corolla, but the other four bird species were nectar robbers. However, an exclusion experiment to eliminate large pollinators has not been used (see Epps *et al.* 2015). Our direct pollinator observations and bird-exclusion experiments in the field demonstrated that birds played an important role in pollination in 10 out of 15 *Rhododendron* species studied in Southwest China. We failed to see birds visiting *R. wardii* but further confirmation needs more efforts. We observed that two or three bird species could act as potential pollinators in one *Rhododendron* species as Georgian *et al.* (2015) observed four bird species visiting *R. floccigerum*, in which Gould’s sunbird (*A. gouldiae*) and dark-backed sibia (*H. melanoleuca*) were shared pollinators for different *Rhododendron* species that were studied here. The winter-flowering *Leucosceptrum canum* with dark-purple nectar was visited by 11 bird species in western Yunnan, China (Zhang *et al.* 2012). Among these 11 birds, the blue-winged minla (*Minla cyanouroptera*) and the oriental white-eye (*Zosterops palpebrosa*) were the most common visitors and were considered to be potential pollinators. These observations might suggest that bird pollinators were not specialists for certain plant species in East Himalayas.

Our investigation of 15 species with diverse floral morphology showed that species with bird pollination tended to occur at higher altitude than insect-pollinated species (Table 1; Huang 2015). Studies have shown that the low temperature is likely to restrict the activity of insects but not birds (see Fang *et al.* 2012; Sun *et al.* 2017) which should be more reliable pollinators at high altitude where low temperature and rain are frequent (Stiles 1971). In *Rhododendron* species with large flowers, successful pollen transfer requires a ‘mechanical fit’ for large animals with the spatial separation of sexual organs from the nectar reward (see Armbruster *et al.* 2011). Previous observations showed that bees were too small to contact the stigma in *Rhododendron* species with relatively large flowers (mismatches between flower and insect morphology) while collecting nectar or pollen (Huang 2015). Visitation by such bees is likely to result in high pollen removal but low pollen receipt as they rarely contact stigmas during pollen collection, an inefficient pollination mode (Wilson and Thomson 1991). In contrast, the foraging behaviour of large animals such as birds, bats and Lepidoptera (butterflies and large moths) could increase the chances of effective pollen transfer through efficient contact with pollen and stigmas when they probe flowers for nectar. We observed that passerine birds perched on the stem below inflorescences of *Rhododendron* species and leaned forward to suck nectar from the base of the corollas. Pollen grains with viscin threads were seen attached to the head or throat of the bird. Compared to most bees, which usually groom pollen, behaviour that contributes much pollen loss during pollen removal, pollen grains of *Rhododendron* species attached to heads or throats of birds and bats or to the wings of Lepidoptera are available for stigma contact, facilitating pollen transfer between flowers.

## Conclusion

Our field observation and pollinator exclusion experiments showed that bird pollination was likely to be involved in 10 of 15 studied *Rhododendron* species in East Himalayas. In cold weather, birds are important pollinators because they forage earlier in the morning than bees (see Kunitake *et al.* 2004; Sun *et al.* 2017). We noted that bird visitation declined after sunrise in our field observations in *Rhododendron* species. Early birds drinking nectar could partly explain why previous observations in the middle of the day did not observe bird pollinators. Birds are likely to evolve higher pollen transfer efficiency than bees because of less grooming and reducing pollen loss during pollen removal, but an experimental test of this hypothesis is strongly needed. The role of other potential pollinators including bats, butterflies and large moths remains to be explored in future studies of *Rhododendron* flowers.

## Sources of Funding

This research was supported National Science Foundation of China (NSFC) grants no. 31730012 to S.-Q.H. and no. 31700196 to Z.-H.H.

## Contributions by the Authors

Z.-H.H. and Y.-P.S. collected data from the field populations. Z.-H.H. and S.-Q.H. wrote the manuscript. All authors contributed in experimental design, data analysis and commented the manuscript.

## Conflicts of Interest

None declared.
